# Iron overload inhibits self‐renewal of human pluripotent stem cells via DNA damage and generation of reactive oxygen species

**DOI:** 10.1002/2211-5463.12811

**Published:** 2020-04-07

**Authors:** Zhenbo Han, Zihang Xu, Lei Chen, Danyu Ye, Yang Yu, Ying Zhang, Yang Cao, Bamba Djibril, Xiaofei Guo, Xinlu Gao, Wenwen Zhang, Meixi Yu, Shenzhen Liu, Gege Yan, Mengyu Jin, Qi Huang, Xiuxiu Wang, Bingjie Hua, Chao Feng, Fan Yang, Wenya Ma, Yu Liu

**Affiliations:** ^1^ Department of Pharmacy The Affiliated Second Hospital Harbin Medical University Harbin China; ^2^ Department of Pharmacology (The Key Laboratory of Cardiovascular Research, Ministry of Education) College of Pharmacy Harbin Medical University Harbin China; ^3^ Beijing Ruihua Heart Rehabilitation Research Center China; ^4^ Department of Clinical Laboratory Fourth Affiliated Hospital of Harbin Medical University China

**Keywords:** cell cycle, DNA damage, human embryonic stem cells, human induced pluripotent stem cells, iron overload, ROS

## Abstract

Iron overload affects the cell cycle of various cell types, but the effect of iron overload on human pluripotent stem cells has not yet been reported. Here, we show that the proliferation capacities of human embryonic stem cells (hESCs) and human induced pluripotent stem cells (hiPSCs) were significantly inhibited by ferric ammonium citrate (FAC) in a concentration‐dependent manner. In addition, deferoxamine protected hESCs/hiPSCs against FAC‐induced cell‐cycle arrest. However, iron overload did not affect pluripotency in hESCs/hiPSCs. Further, treatment of hiPSCs with FAC resulted in excess reactive oxygen species production and DNA damage. Collectively, our findings provide new insights into the role of iron homeostasis in the maintenance of self‐renewal in human pluripotent stem cells.

## Graphical abstract

We demonstrated in this study that iron overload could severely impair the proliferation of human embryonic stem cells/human induced pluripotent stem cells through excess reactive oxygen species production and DNA damage. These results imply a role of iron homeostasis in maintaining the self‐renewal of human embryonic stem cells/human induced pluripotent stem cells.

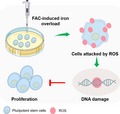

**Abbreviations**
ALP: alkaline phosphatase
DFO: deferoxamine
EdU: 5‐ethynyl‐20‐deoxyuridine
FAC: ferric ammonium citrate
hESC: human embryonic stem cell
hiPSC: human induced pluripotent stem cell
hPSC: human pluripotent stem cell
PFA: paraformaldehyde
PSC: pluripotent stem cell
ROS: reactive oxygen species
SEM: standard error of the mean

Embryonic stem cells (ESCs) and induced pluripotent stem cells (iPSCs) have the ability to self‐renew and differentiate into almost all types of somatic cells *in vitro* [1, 2]. Therefore, ESCs and iPSCs could be used for a broad range of clinical applications in regenerative medicine [3, 4, 5, 6]. It has been reported that genetic and epigenetic networks synergistically maintain the self‐renewal status of human embryonic stem cells (hESCs)/human induced pluripotent stem cells (hiPSCs) [7, 8]. However, the details of the mechanism of self‐renewal maintenance in PSCs are still not yet fully understood.

Iron is one of the most essential microelements in the body. However, it has been reported that excess iron can initiate some signaling pathways that are essential for cell death because it has the ability of the redox activity and can produce reactive oxygen species (ROS) [9, 10]. In recent years, numerous studies have revealed that unbalanced iron homeostasis was closely connected with certain common human diseases, such as anemia of chronic disease and obesity [11, 12, 13]. In addition, many studies have reported that iron homeostasis can influence the functions of some kinds of stem cells. For example, it has been clarified that specific targeting of iron homeostasis in cancer stem cells can increase the cancer therapy [14]. Besides, our group discovered that iron deficiency caused inhibition of pluripotency in hESCs/hiPSCs [15]. However, the effect of iron overload in iPSCs or ESCs has not yet been reported.

Here, we studied the function of iron overload in human pluripotent stem cells (hPSCs). We investigated that iron overload inhibits the proliferation of hPSCs through excess ROS production and DNA damage. Our study provides novel insights into the maintenance of hPSC self‐renewal.

## Materials and methods

### Cell culture

Human ESCs (H1‐ESCs) and iPSCs (AC‐iPSCs) were cultured as described in our previous study [15]. In brief, cells were cultured in E8 pluripotent stem cell culture medium (STEMCELL Technology, Vancouver, British Columbia, Canada) at a temperature of 37 °C in 5% CO_2_. When the cell density reached 70%, we subcultured the cells with EDTA acid (CELLAPY, Beijing, China).

### Quantitative real‐time PCR

A quantitative real‐time PCR assay was performed as described previously [16]. Total RNA was isolated using TRIzol reagent (Ambion, Life Technologies, Camarillo, CA, USA) according to the manufacturer’s protocol. The FastStart Universal SYBR Green Master (Rox) and 7500 Fast Real‐Time PCR System (Applied Biosystems, Foster City, CA, USA) were used in real‐time PCR for relative quantification of RNA. Every sample was tested four times. The primers are listed in Table S1.

### Alkaline phosphatase staining

A 5‐bromo‐4‐chloro‐3‐indolyl phosphate/Nitro Blue tetrazolium/alkaline phosphatase (ALP) color development kit (C3206; Beyotime, Nantong, Jiangsu, China) was used for ALP staining. In brief, 4% paraformaldehyde (PFA) was used to immobilize cells for 30 min at 37 °C. The cells were then washed three times in Dulbecco’s PBS. Then, cells were incubated in the dark in 5‐bromo‐4‐chloro‐3‐indolyl phosphate/Nitro Blue tetrazolium solution for 7 min. Distilled water was immediately added to end the reaction. Cells were photographed under a standard light microscope (Eclipse TS100; Nikon, Melville, NY, USA).

### 5‐Ethynyl‐20‐deoxyuridine assay

The effect of iron overload on cell proliferation was determined by 5‐ethynyl‐20‐deoxyuridine (EdU) assay. Cells were cultured in glass‐bottomed cell culture dishes. Then, 4% PFA was used to immobilize cells for 15 min and after 0.4% Triton X‐100 was used to permeabilize the cells for 20 min. Then, a cell proliferation assay was carried out using an EdU assay kit (Ribobio Co., Ltd., Guangzhou, China). The cells were photographed with a confocal fluorescence microscope (FV10C‐W3; Olympus, Tokyo, Japan). The cell nuclei of double‐labeled EdU and 4′,6‐diamidino‐2‐phenylindole (DAPI) were considered markers of positive cells.

### Cellular iron content assay

An iron assay kit (K390‐100; BioVision, Milpitas, CA, USA) was used to detect cellular iron content. First, cells were lysed with 120 μL iron assay buffer (K3900‐100‐1; BioVision). Subsequently, the liquid was collected and centrifuged at 16 000 ***g*** for 10 min. Then, it was added along with 35 μL sample and 65 μL assay buffer to 96‐well plates, and 5 μL iron reducer (K3900‐100‐3; BioVision) was added to each well. The cells were incubated at 25 °C for 30 min. Next, 100 μL iron probe (K3900‐100‐2; BioVision) was added, and the solution was mixed and then incubated in the dark at 25 °C for 30 min. The absorbance was measured at 593 nm using a microplate reader.

### Western blotting

Cells were lysed with radioimmunoprecipitation assay (RIPA) buffer (P0013; Beyotime). Subsequently, proteins were separated via 12.5% SDS/PAGE and transferred to a nitrocellulose blotting membrane (Life Science, Mexico Assembly). The membrane was blocked and then incubated with primary antibodies overnight at 4 °C. It was then incubated with secondary antibody for 1 h at room temperature. The membrane was then washed three times with PBST and examined using odyssey version 1.2 software (LI‐COR Biosciences, Lincoln, NE, USA). The antibodies are listed in Table S2.

### Measurement of ROS production

Intracellular levels of ROS production were measured using a ROS Assay Kit (S0033; Beyotime Biotechnology, Shanghai, China) according to the manufacturer’s instructions. In brief, after washing cells with PBS, cells were incubated with 10 μm 2′,7′‐Dichlorodihydrofluorescein diacetate (DCFH‐DA) probes for 30 min at 37 °C. Then, the cells were fixed in 4% PFA for 30 min and stained with DAPI (20 μg·mL^−1^) for 10 min. The cells were then imaged with a fluorescence microscope (Olympus Optical).

### Immunofluorescence assays

Cells were fixed with 4% PFA for 15 min at 37 °C. Then, cells were washed with PBS and penetrated with 0.3% Triton X‐100 for 15 min. Next, cells were blocked with goat serum. After, the cells were stained with ROS overnight. Then, the cells were stained with secondary antibody for 1 h. Cell nuclei were stained with DAPI. The cells were then observed under a confocal fluorescence microscope (FV10C‐W3; Olympus). The antibodies are listed in Table S2.

### Statistics

Error bars represent the standard error of the mean (SEM). Data were analyzed using prism 7 (GraphPad Software Inc., San Diego, CA, USA). The significance of differences was analyzed using one‐way ANOVA and presented with the following levels of significance: *P < 0.05, **P < 0.01 and ***P < 0.005.

## Results

### Iron overload inhibits self‐renewal of human PSCs

**Fig. 1:**
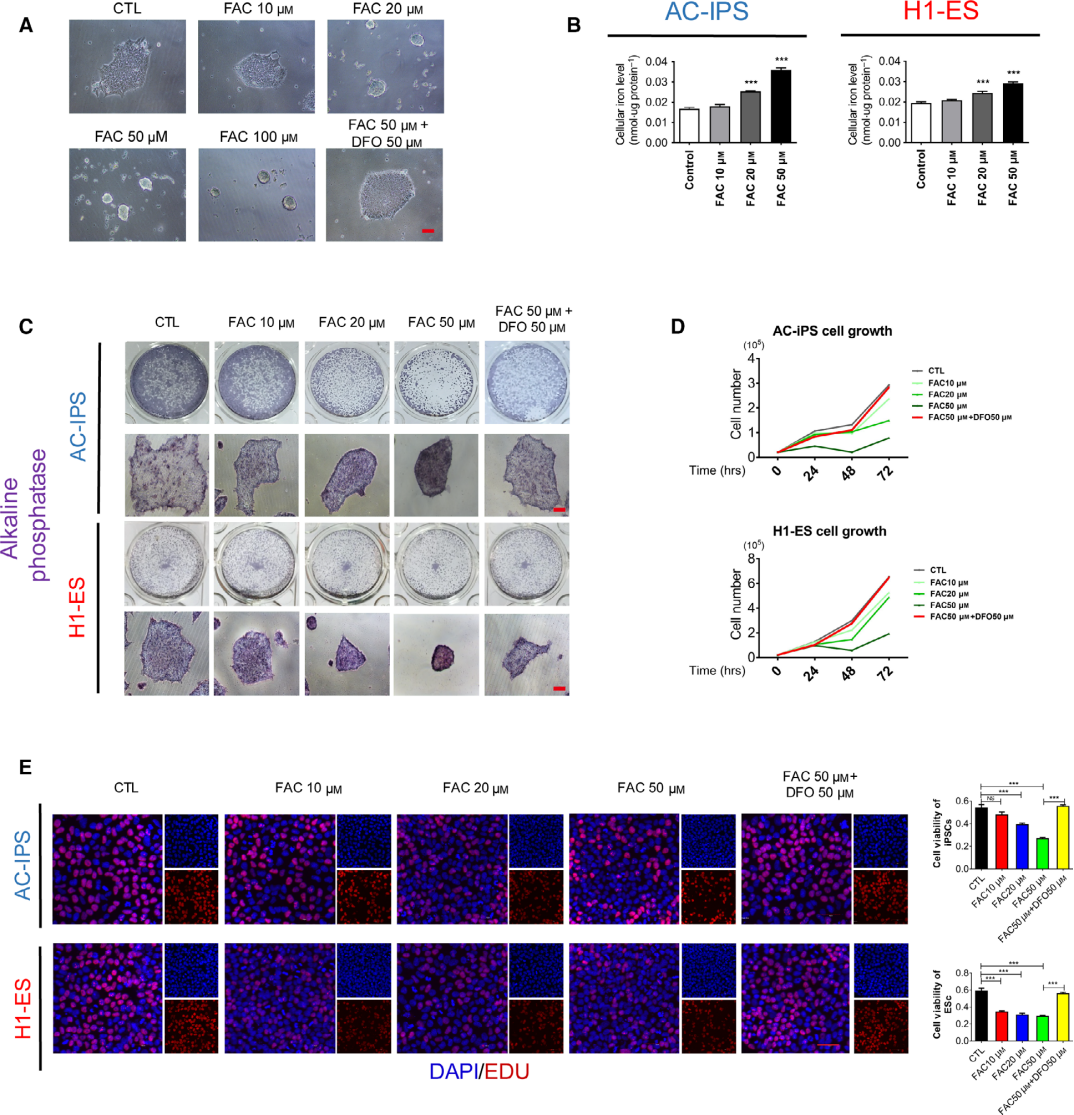
(A) Phase images of hiPSCs treated with 10, 20, 50 or 100 μm FAC or 50 μm FAC + 50 μm DFO. Scale bar: 100 μm. (B) The cellular iron content of AC‐iPSCs and H1‐ESCs treated with different concentrations of FAC for 16 h. Data are shown as mean ± SEM (*n* = 3). One‐way ANOVA was applied. ***P < 0.001 compared with the control. (C) Morphology and ALP staining of hiPSCs/hESCs treated with different concentrations of FAC or 50 μm DFO supplemented with 50 μm FAC for 16 h. Scale bars: 100 μm. (D) Cell counting assay of AC‐iPSCs and H1‐ESCs supplemented with 10, 20 or 50 μm FAC or 50 μm FAC + 50 μm DFO. (E) EdU assay of hiPSCs/hESCs treated with different concentrations of FAC or 50 μm DFO supplemented with 50 μm FAC for 16 h. Scale bar, 50 μm. Data are shown as mean ± SEM (*n* = 3). One‐way ANOVA was applied. ***P < 0.001.

To characterize the role of iron overload in human PSCs, we treated hESCs/hiPSCs with different concentrations (0, 10, 20 and 50 μm) of ferric ammonium citrate (FAC) and 50 μm iron chelator deferoxamine (DFO). Interestingly, we observed that FAC‐treated cells exhibited a dramatically slower rate of proliferation compared with the control group (Fig. 1A). We first measured cellular iron content after FAC treatment. The results showed that the iron content of the hPSCs was increased by FAC treatment in a concentration‐dependent manner (Fig. 1B). As shown by ALP staining, iron overload significantly inhibited the proliferation of hESCs/hiPSCs, but cells treated with FAC were maintained with undifferentiated colony morphology (Fig. 1C). The cell counting assay also showed that the proliferation of hESCs/hiPSCs was significantly inhibited after FAC treatment. However, DFO 50 μm protected hESCs/hiPSCs from cell‐cycle arrest induced by FAC 50 μm (Fig. 1D). Meanwhile, we used an EdU incorporation assay to assess cell proliferation capacity. As expected, there were more EdU‐positive cells in the control group than in the FAC group (Fig. 1E). Together, these results indicate that iron overload inhibited self‐renewal of hPSCs.

### Iron overload did not affect pluripotency in hPSCs

**Fig. 2:**
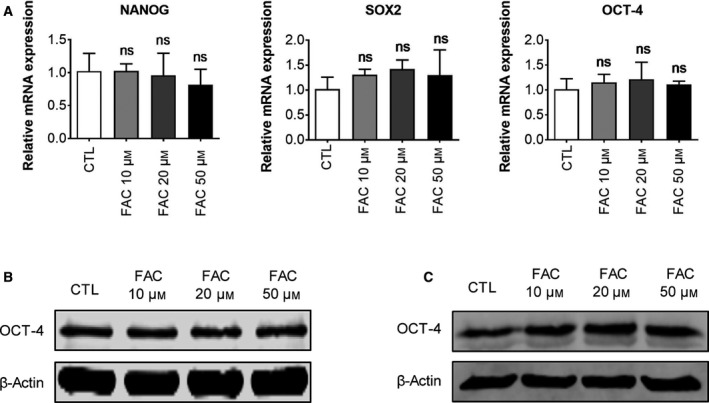
(A) Quantitative RT‐PCR analysis of NANOG, SOX2 and OCT‐4 expression in AC‐iPSCs treated with different concentrations of FAC for 16 h. Data are shown as mean ± SEM (*n* = 3). One‐way ANOVA was applied. (B) Western blot analysis of OCT‐4 expression in AC‐iPSCs treated with different concentrations of FAC for 16 h. (C) Western blot analysis of OCT‐4 expression in H1‐ESCs treated with different concentrations of FAC for 16 h.

We next investigated whether iron overload affected the pluripotency of hPSCs. We performed quantitative RT‐PCR analysis to detect the mRNA expression of pluripotency factors in hiPSCs after exposure to different concentrations of FAC for 16 h. The results show that after treatment with FAC, hiPSCs exhibited similar expression of pluripotency factors to the control group (Fig. 2A). This finding was also supported by our western blot analysis (Fig. 2B,C). The earlier results suggest that iron overload inhibits the proliferation but has no influence on the pluripotency of hESCs/hiPSCs.

### Increased intracellular ROS levels in hPSCs after FAC treatment

**Fig. 3:**
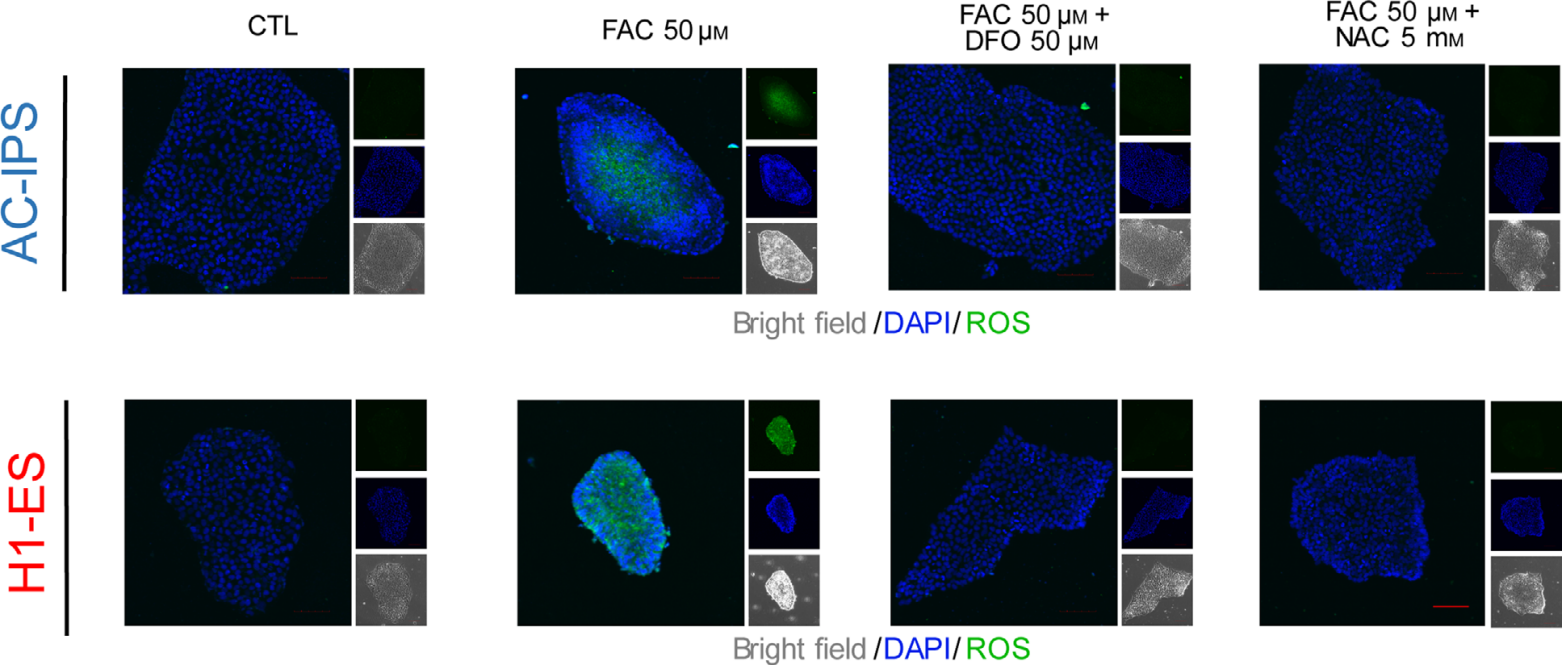
DCFH‐DA staining of AC‐iPSCs and H1‐ESCs treated with 50 μm FAC, 50 μm FAC + 50 μm DFO and 50 μm FAC + 5 mm NAC. Scale bar, 100 μm.

Next, we focused on exploring the mechanism by which iron overload regulates self‐renewal in hPSCs. Intracellular ROS levels play crucial roles in the proliferation of various cell types. It has been reported that excess ROS generation is involved in iron dyshomeostasis‐induced cell injury [17, 18, 19]. To further evaluate the effects of iron overload on ROS production, we performed DCFH‐DA staining in hPSCs after exposure to different concentrations of FAC. As shown in Fig. 3, FAC 50 μm significantly induced ROS production in hiPSCs. Meanwhile, 50 μm DFO and 5 mm ROS scavenger N‐acetyl‐L‐cysteine (NAC) suppressed FAC‐induced ROS production. In addition, we found that DFO‐induced iron deficiency also increased ROS production in hiPSCs (Fig. S1). These data indicate that iron dyshomeostasis resulted in a significant increase in ROS in hPSCs.

### Iron overload leads to DNA damage in hESCs/hiPSCs

**Fig. 4:**
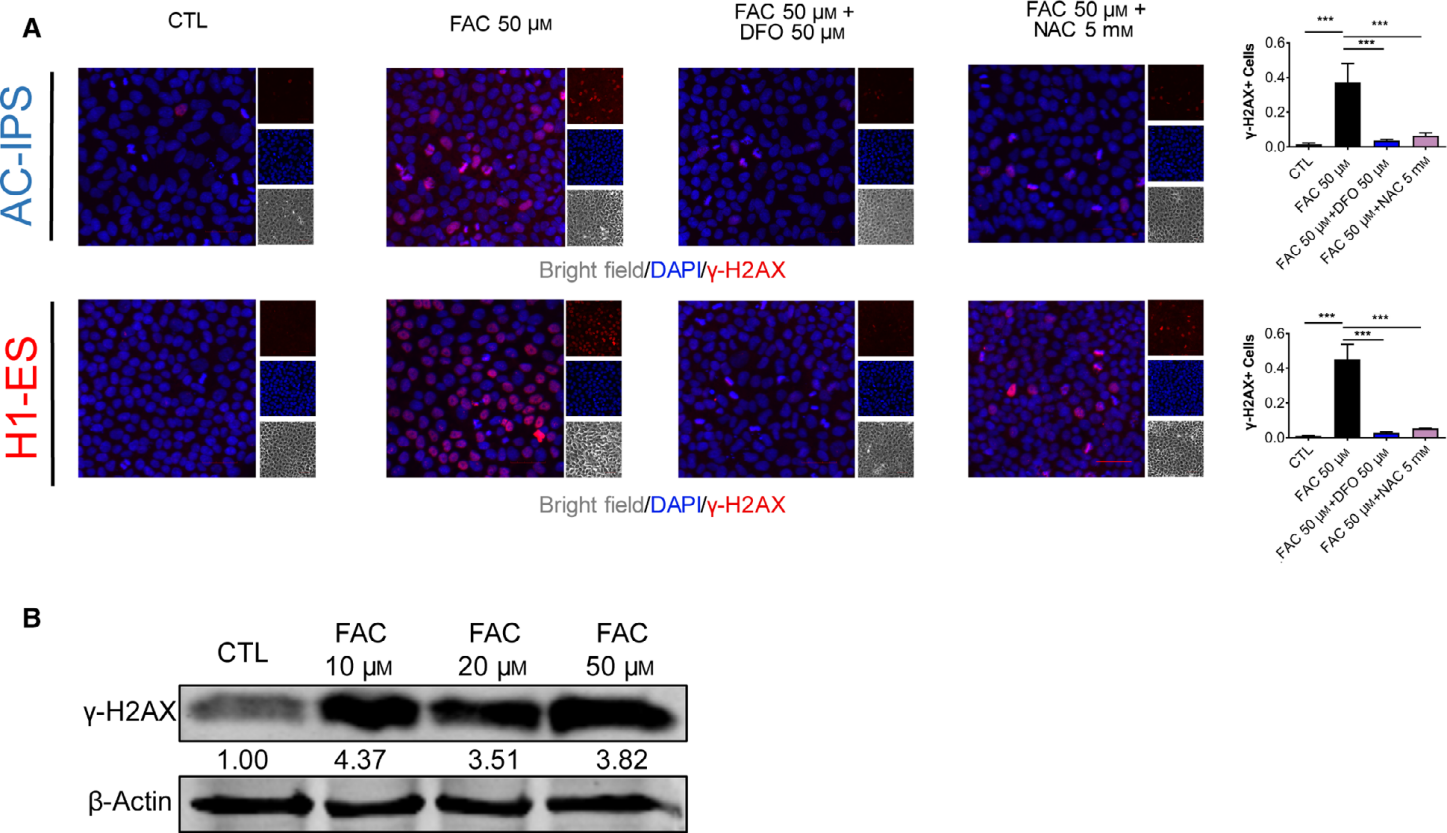
(A) Immunofluorescence analysis of DNA damage in AC‐iPSCs and H1‐ESCs treated with 50 μm FAC, 50 μm FAC + 50 μm DFO and 50 μm FAC + 5 mm NAC. Scale bars, 50 μm. Data are shown as mean ± SEM (*n* = 4). One‐way ANOVA was applied. ***P < 0.001. (B) Western blot analysis of γ‐H2A.X expression in H1‐ESCs treated with different concentrations of FAC for 16 h.

Previous studies have indicated that increased ROS production resulted in DNA damage [20]. We thus examined whether iron overload causes DNA damage in hiPSCs and hESCs. We stained cells with γ‐H2A.X (phosphorylation status of H2A.X), a marker of DNA damage. As shown in Fig. 4A, after exposure to FAC for 16 h, iPSCs/ESCs exhibited a significant increase in DNA damage. However, DFO and NAC treatment suppressed the increase of γ‐H2A.X‐positive cells upon FAC treatment. The protein expression of γ‐H2A.X also increased after treatment with different concentrations of FAC (Fig. 4B). These results indicate that iron overload results in a sharp increase in DNA damage in hPSCs.

## Discussion

This study reveals that iron overload inhibited the proliferation of hESCs/iPSCs without affecting pluripotency. The underlying mechanisms of this phenomenon were associated with DNA damage and excessive ROS production. These findings provide a better understanding of the maintenance of self‐renewal in hPSCs.

Iron overload is mainly caused by certain genetic diseases and high‐iron diets. Excessive iron accumulation in the body can cause liver damage, cardiovascular damage, neurodegenerative diseases and many aging‐related diseases [21]. Iron overload has a profound effect on immature hematopoietic cells and stromal cells, and thereby destroys the hematopoietic process [22]. Previous studies have indicated that iron homeostasis is crucial for various kinds of stem cell. For example, iron overload can impact the self‐renewal and function of hematopoietic stem cells and progenitor cells [23, 24], whereas iron deficiency impairs the pluripotency of hESCs/hiPSCs [15]. Besides, it has been reported that iron deficiency inhibited the expression of stemness markers and spherogenesis in cancer stem cells [25]. However, our findings indicate that iron overload can restrict self‐renewal in hPSCs via excessive ROS production and DNA damage, which provides a new perspective for studying the role of iron homeostasis in the maintenance of hPSC self‐renewal.

hESCs and iPSCs have the ability to self‐renew and differentiate into all cell types. Therefore, human PSCs have emerged as a promising cell resource for regenerative medicine and a particular experimental model [26]. Accumulated evidence from studies of hESCs suggests that the self‐renewal of ESCs was regulated via ROS to a certain degree [27, 28]. Meanwhile, a previous study indicated that stem cells were highly susceptible to DNA damage and cause DNA damage accumulation in the microenvironment of growing stem cells, which was partly responsible for catastrophic consequences for tissue and body homeostasis [29]. For example, the accumulation of DNA damage affected the genomic stability of hematopoietic stem cells and may increase the incidence of hematological malignancies, especially with age [30, 31]. The production of ROS is also one of the reasons for the accumulation of DNA damage. Although our study revealed that hPSC proliferation was inhibited because iron overload caused excessive ROS production and DNA damage, the molecular mechanism of cell proliferation induction requires further study.

In summary, our study shows that iron overload inhibited self‐renewal capacity of hESCs and hiPSCs, which provides a new insight into the potential mechanisms of the self‐renewal of PSCs.

## Conflict of interest

The authors declare no conflict of interest.

## Author contributions

ZH, WM and YL conceived the study and designed the experiments. ZH, ZX, LC, DY, YY, YZ, YC, DB, X. Gao, X. Guo, WZ, MY, SL, GY, MJ, QH, XW, BH, CF and FY contributed to the data collection, performed the data analysis and interpreted the results. ZH wrote the manuscript. WM and YL contributed to the critical revision of this article. All authors read and approved the final manuscript.

## Supporting information


**Fig. S1** **.** DFO‐induced iron deficiency promotes ROS generation in hiPSCs. DCFH‐DA staining of AC‐IPSCs treated with 150 μm DFO for 24 h. Scale bar, 100 μm.
**Table S1** **.** Primers used in quantitative RT‐PCR.
**Table S2** **.** Antibodies used in this study.
