# Supplementary material for: Iron overload inhibits self‐renewal of human pluripotent stem cells via DNA damage and generation of reactive oxygen species
Source: FEBS Open Bio. 2020 Apr 7;10(5):726–33. doi: 10.1002/2211-5463.12811 (PMC7193162; doi:10.1002/2211-5463.12811)
Supplement: Supplementary file 1 — Fig. S1 . DFO‐induced iron deficiency promotes ROS generation in hiPSCs. DCFH‐DA staining of AC‐IPSCs treated with 150 μm DFO for 24 h. Scale bar, 100 μm. Table S1 . Primers used in quantitative RT‐PCR. Table S2 . Antibodies used in this study. [file FEB4-10-726-s001.docx]

**Supplementary Information**

**Iron Overload inhibits Self Renewal of Human Pluripotent Stem Cells Via DNA Damage and Reactive Oxygen Species Generation**

Zhenbo Han^2*^, Zihang Xu^2*^, Lei Chen^3*^, Danyu Ye^2^, Yang Yu^2^, Ying Zhang^2^, Yang Cao^2^, Djibril Bamba^2^, Xiaofei Guo^2^, Xinlu Gao^2^, Wenwen Zhang^2^, Meixi Yu^2^, Shenzhen Liu^2^, Gege Yan^2^, Mengyu Jin^2^, Qi Huang^2^, Xiuxiu Wang^2^, Bingjie Hua^2^, Chao Feng^2^, Fan Yang^2^, Wenya Ma^2*^, Yu Liu^1*^

**Supplementary Figures**

Fig. S1


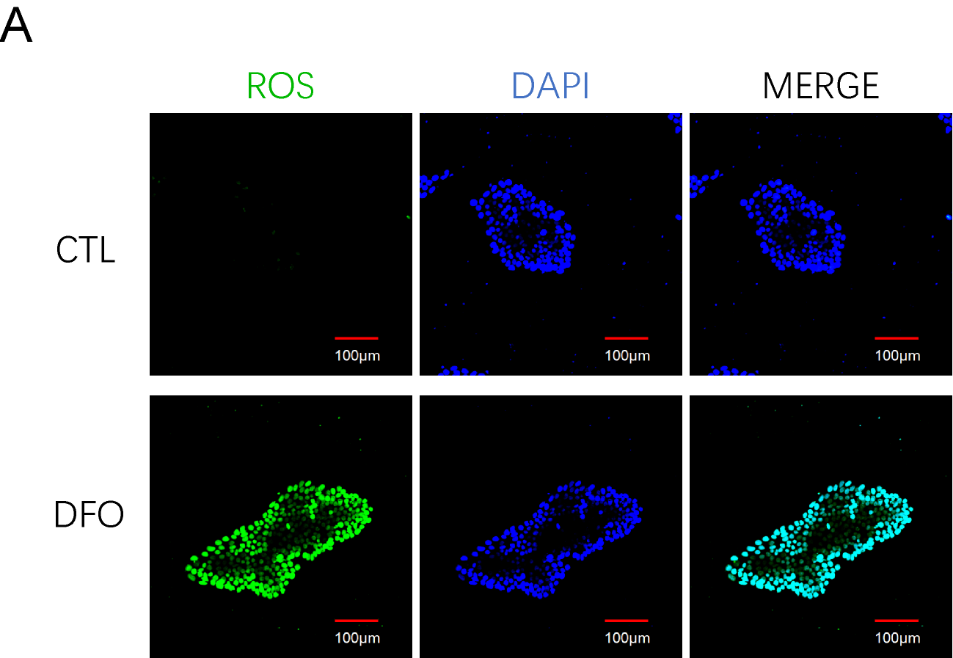


**Supplementary Figure legends**

**Figure S1. DFO-induced iron deficiency promotes ROS generation in hiPSCs.**

**A.** DCFH-DA staining of AC-IPS cells treated with 150 μM DFO for 24 h. Scale bar, 100 μm.

**Supplementary Table 1: Primers used in RT-qPCR.**

|  | Gene | Forward 5’-3’ | Reverse 5'-3’ |
| --- | --- | --- | --- |
| 1 | NANOG | ACCTATGCCTGTGATTTGTGG | AGTGGGTTGTTTGCCTTTGG |
| 2 | SOX-2 | GGTTACCTCTTCCTCCCACTCC | CCCTCCCATTTCCCTCGTTT |
| 3 | OCT-4 | ACCTGGAGTTTGTGCCAGGGTT | TTCCCTCCAACCAGTTGCCCCA |
| 4 | 18s | CCTGGATACCGCAGCTAGGA | GCGGCGCAATACGAATGCCC |

| Antibodies | Source | Cat. No |
| --- | --- | --- |
| Oct-4 | Santa Cruz | SC-5279 |
| γ-H2AX | Abcam | ab26350 |
| β-ACTIN | ZHONG SHAN JIN QIAO | TA-09 |

**Supplementary Table 2: Antibodies used in this study**
